# Radiation and CAR T-cell Therapy in Lymphoma: Future Frontiers and Potential Opportunities for Synergy

**DOI:** 10.3389/fonc.2021.648655

**Published:** 2021-03-25

**Authors:** Penny Q. Fang, Jillian R. Gunther, Susan Y. Wu, Bouthaina S. Dabaja, Loretta J. Nastoupil, Sairah Ahmed, Sattva S. Neelapu, Chelsea C. Pinnix

**Affiliations:** ^1^Department of Radiation Oncology, The University of Texas MD Anderson Cancer Center, Houston, TX, United States; ^2^Department of Lymphoma/Myeloma, The University of Texas MD Anderson Cancer Center, Houston, TX, United States

**Keywords:** chimeric antigen receptor T cells, radiation therapy, large B cell lymphoma, immunotherapy, external beam irradiation

## Abstract

CAR T-cell therapy has revolutionized the treatment approach to patients with relapsed/refractory hematologic malignancies; however, there continues to be opportunity for improvement in treatment toxicity as well as response durability. Radiation therapy can play an important role in combined modality treatments for some patients undergoing CAR T-cell therapy in various clinical settings. In this review, we discuss the current evidence for RT in the setting of CAR T-cell therapy for patients with hematologic malignancies and propose potential opportunities for future investigation of RT and CAR T-cell treatment synergy. Future research frontiers include investigation of hypotheses including radiation priming of CAR T-cell mediated death, pre-CAR T-cell tumor debulking with radiation therapy, and selection of high risk patients for early radiation salvage after CAR T cell therapy.

## Introduction

Chimeric antigen receptor (CAR) T-cell therapy has transformed our approach to patients with relapsed/refractory (R/R) aggressive lymphomas, with multiple therapies that have achieved high response rates and notable durable disease remissions in patients with otherwise dismal outcomes. Radiation therapy (RT) may be a valuable treatment modality that, when optimally combined with CAR T-cell therapy, could offer enhanced tumor control and reduced toxicity. In this review, we discuss the current evidence for RT in the setting of CAR T-cell therapy for patients with hematologic malignancies and propose potential opportunities for future investigation of RT and CAR T-cell treatment synergy.

## Background

While most patients with diffuse large B-cell lymphoma (DLBCL) respond to frontline immunochemotherapy based regimens [typically rituximab, cyclophosphamide, doxorubicin, vincristine, and prednisone (R-CHOP)], roughly 30–40% of patients are refractory to primary therapy or develop relapsed disease (1, 2). Until recently, the primary potentially curative salvage therapy approach included multi-agent platinum-based chemotherapy followed by high dose chemotherapy and autologous stem cell transplantation (3–5), with an associated overall response rate (ORR) of roughly 60% and 3-year overall survival (OS) of roughly 50%. However, for patients that do not respond to second line therapy, median survival is exceptionally poor at roughly 4–6 months (6, 7). In a large, international multicohort retrospective study of patients with relapsed and refractory DLBCL, only 20% of patients were alive at 2 years (8).

CD-19 CAR T-cell therapy has ushered in a new era of therapeutic approaches for patients with R/R large B-cell lymphoma (9). Autologous T-cells are genetically engineered to express chimeric antigen reception molecules that target the CD-19 antigen on the surface of large B-cell lymphoma cells. Patients are administered lymphodepleting conditioning chemotherapy, most commonly fludarabine and cyclophosphamide, over 3 days prior to infusion of the autologous CAR T-cell product.

Autologous anti-CD19 CAR T-cell therapy with axicabtagene ciloleucel (axi-cel) induced ORR and complete response (CR) rates of 83 and 58%, respectively, among patients with R/R large B-cell lymphoma in the multicenter ZUMA-1 trial; responses were sustained among 39% of patients with a median follow up time of ~27 months (10, 11). Based on these results, axi-cel was approved by the Federal Drug Administration (FDA) in October 2017 for R/R DLBCL, transformed follicular lymphoma, primary mediastinal large B-cell lymphoma, and high-grade B-cell lymphoma. Tisagenlecleucel (tisa-cel) was subsequently FDA approved for patients with large B-cell lymphoma based on the results from the JULIET trial demonstrating an ORR of 52% and CR rate of 40%; ongoing response at 6 months was observed in 33% of patients (12, 13). The TRANSCEND multicenter trial enrolled 344 patients with R/R large B-cell lymphomas who underwent apheresis for the production of lisocabtagene maraleucel (liso-cel). Among the patients included in the efficacy evaluation, the ORR was 73% and the CR rate was 53% (14). FDA approval for liso-cel is pending. The FDA most recently approved the first CAR T-cell product for adults with R/R mantle cell lymphoma, brexucabtagene autoleucel (bruxa-cel), based on the promising results of the ZUMA-2 trial which demonstrated responses in 93% of patients and CR in 67% (15). Roughly 57% of patients had sustained responses with a median follow up of 12.3 months (15). CAR T-cell therapy is undergoing active investigation in nearly all hematologic malignancies, with promising results emerging in Hodgkin lymphoma (16), multiple myeloma (17), and follicular lymphoma (18–20), among others.

While the high ORRs and notable proportion of patients achieving durable responses have been encouraging, there continues to be opportunity for improvement in treatment toxicity as well as response durability. Radiation therapy is a potential tool that, when coupled with CAR T-cell therapy, may offer the opportunity to improve outcomes.

Mechanistically, there is early evidence of potential synergy between radiation and CAR T-cell therapy, which provides additional impetus for investigation into their combined use. Potential complementary pathways that have been identified are mediated by effect of radiation on the tumor-microenvironment or in priming the local or systemic immune response. For example, preclinical studies show that low dose RT conditioning sensitizes antigen-negative tumor cells to CAR T-mediated apoptosis by making tumor cells susceptible to tumor necrosis factor-related apoptosis-inducing ligand (TRAIL)-mediated death (21). RT also enhances cytotoxic T-cell migration to irradiated areas, reverses T-cell exhaustion, and diversifies the T-cell receptor repertoire of tumor infiltrating lymphocytes (22). RT has complementary immunomodulatory activity through induction of increased major histocompatibility complex (MHC)-1 expression and liberation of antigens on irradiated cells, producing enhanced tumor-specific immunity via epitope spreading against irradiated and distant sites (23). In the following sections, we will discuss the potential role of radiation in CAR T-cell therapy with respect to the time at which radiation is administered relative to apheresis and CAR T-cell infusion. We propose that radiation therapy may have an important future role in tumor debulking, pre-infusion conditioning, and post-infusion rescue of residual or resistant disease.

## Radiation as Bridging Therapy, Between Apheresis and Car T Infusion

During the period of CAR T-cell manufacturing, typically 3–4 weeks at minimum, patients may require bridging therapy to maintain control of disease and avoid the morbidity of symptomatic disease progression. Many of the initial clinical trials did not allow bridging therapy, however, in practice many patients require therapy for disease control prior to infusion of CAR T-cells. The optimal therapeutic regimen for bridging depends on the patient's treatment history and prior toxicities, however ideally enough time should be allowed between bridging and CAR T-cell infusion—a washout period—so as to allow recovery from adverse events, particularly if there is overlapping toxicity that may prompt treatment with steroids, which may blunt the CAR-T response.

Bridging therapy can include steroids for symptom control, radiation, chemotherapy or a combination. The results from several studies provide early evidence that RT as a bridging treatment can be safe and effective (Table 1). In an initial published report of RT as bridging prior to CD-19 CAR T-cell therapy from Moffitt Cancer Center by Sim et al. 12 patients were intended for RT bridging prior to axi-cel therapy (24). RT was initiated after apheresis in most patients (n = 10, 83%). Concurrent systemic therapy was administered to 7 patients (58%). Eleven patients went on to receive an axi-cel infusion. At a median follow up of 3.3 months, the ORR was 81.8% and CR was achieved in 45% (5 of 11 patients). Severe CAR T-cell toxicity defined as grade 3 or higher cytokine release syndrome (CRS) or immune effector cell-associated neurotoxicity syndrome [ICANS, previously termed CAR-T-cell-related encephalopathy syndrome (CRES)] occurred in 3 of 11 patients, consistent with the rates of this complication in the larger prospective studies. The authors also evaluated serum blood counts and observed neutropenia in 1/3 of patients after RT. White blood cell count and absolute lymphocyte counts also decreased slightly with RT. Anemia, thrombocytopenia, and neutropenia are also common after lymphodepleting conditioning therapy so the contribution of RT to these cytopenias is unclear. Ultimately this initial report demonstrated the safety and feasibility of an RT bridging approach.

**Table 1:** Characteristics and results of initial reports of radiation therapy as bridging treatment prior to CD-19 CAR T-cell therapy among non-Hodgkin lymphoma patients.

**Study**	**Moffitt [Sim et al. (24)]**	**MDACC [Pinnix et al. (25)]**	**UPenn [Wright et al. (27)]**
Patient population	R/R DLBCL, tFL	R/R DLBCL, tFL, PMBCL	R/R aggressive B-cell lymphoma
Product	Axi-cel	Axi-cel	Axi-cel (*n* = 18), tisa-cel (*n* = 13)
Total # Pts apheresed	12	148	31
Received CAR T, %	92% (*n* = 11)	84% (*n* = 124)	100% (*n* = 31)
Received bridging therapy, %	100% (*n* = 12)	50% (*n* = 62)	NR
**Received RT bridging (%)**			
RT alone, %	42% (*n* = 5)	65% (*n* = 11)	60% (*n* = 3)
CMT, %	58% (*n* = 7)	35% (*n* = 6)	40% (*n* = 2)
Median RT dose, Gy (range)	20 Gy (6–30)	35 Gy (9–46)	37.5 Gy (20–45)
RT fraction range	2–4 Gy	1.8–5 Gy	2.2–4 Gy
**Timing of RT bridging**			
Before apheresis, %	17% (*n* = 2)	35% (*n* = 6)	NR
After apheresis, %	83% (*n* = 10)	65% (*n* = 11)	
**RT field size**			
Comprehensive, %	42% (*n* = 5)	53% (*n* = 9)	60% (*n* = 3)
Focal, %	58% (*n* = 7)	47% (*n* = 8)	40% (*n* = 2)
CRS grade ≥3, %	8% (*n* = 1)	6% (*n* = 1)	0% (*n* = 0)
ICANS grade ≥3, %	25% (*n* = 3)	35% (*n* = 6)	0% (*n* = 0)
Median follow up time (range)	3.3 months (1.1–12)	11.1 months (95% CI 9.9–12.3)	12.3 months (9.8–19.9)
ORR	81.8%	100% for RT alone, 67% for CMT	80%
CR rate	45.5%	82% for RT alone, 67% for CMT	60%
1-year PFS	NR	44% RT alone, 25% CMT	20%
1-year OS	NR	63% RT alone, 25% CMT	80%

*R/R, relapsed and refractory; DLBCL, diffuse large B-cell lymphoma; tFL, transformed follicular lymphoma, PMBCL, primary mediastinal B-cell lymphoma; axi-cel, axicabtagene ciloleucel; tisa-cel, tisagenlecleucel; Pts, patients, CAR T, chimeric antigen receptor T cell therapy; RT, radiation therapy; NR, not reported; CMT, combined modality therapy; Gy, gray; CRS, cytokine release syndrome; ICANS, immune effector cell-associated neurotoxicity syndrome; CI, confidence interval; ORR, overall response rate; CR, complete response; PFS, progression free survival; OS, overall survival*.

In a retrospective series from MD Anderson Cancer Center (MDACC), the impact of bridging therapy was evaluated among 148 patients with R/R large B-cell lymphoma who underwent apheresis with the intention of delivering commercially available axi-cel therapy (25). In this study 16% of patients (*n* = 24) did not receive axi-cel therapy mainly due to progressive lymphoma. Among the 124 patients that received axi-cel therapy, 50% received bridging therapy including RT alone (*n* = 11), RT combined with systemic therapy (*n* = 6), or systemic therapy alone (*n* = 45). For all patients that received RT (*n* = 17), the median RT dose was 35 Gy. RT was administered after leukapheresis in 65% of patients (*n* = 11). In this study there was no difference in grade 3 or higher CRS or ICANS between any of the bridging or non-bridging cohorts. Interestingly, there was a trend toward decreased 1-year progression free survival (PFS) among patients who received any type of bridging therapy, at 29% compared to 44% in those who did not receive bridging treatment (*p* = 0.06). It is important to note, however, that patients who received bridging therapy (n=62) were more likely to have poor prognostic features at the time of apheresis such as Eastern Cooperative Oncology Group (ECOG) performance status of 2–3, international prognostic index (IPI) score of 3 or greater, bulky disease (defined as 10 cm or greater), and elevated lactate dehydrogenase (LDH). These characteristics have been shown to be associated with inferior survival outcomes in a large retrospective multicenter US Lymphoma CAR T Consortium study of R/R LBCL patients treated with standard of care axi-cel therapy (26). Therefore, it is unclear if bridging therapy itself is associated with shorter PFS or if confounding patient and disease factors are significantly contributing.

In the MDACC study, patients bridged with RT alone had a 1-year PFS of 44%, which was comparable to patients that did not receive bridging therapy (1-year PFS of 44%, *p* = 0.52). Both the cohort of patients bridged with RT combined with systemic therapy and the cohort bridged with systemic therapy alone had a 1-year PFS of 25%. The ORR and CR rates were higher for the patients that received single modality RT bridging at 100 and 82%, respectively, which were significantly higher than the ORR and CR rates for the systemic therapy alone cohort (67% ORR, *p* = 0.03 and 38% CR rate, *p* = 0.01), and compared favorably with the non-bridged cohort (82% ORR, *p* = 0.13 and 48% CR rate, *p* = 0.04). Taken together, this study demonstrated the efficacy of single modality RT as an effective bridging option for disease control prior to CAR T-cell therapy.

Similarly, Wright et al. conducted a retrospective study of 31 patients receiving tisa-cel or axi-cel for R/R aggressive B-cell lymphoma, of which 5 patients received bridging RT with a median RT dose of 37.5 Gy within 30 days of CAR T infusion (27). The study also included 26 patients that received non-bridging RT (delivered more than 30 days prior to CAR T infusion) or had no prior RT. No patients in the bridging RT group experienced grade 3 or higher CAR T related CRS or ICANS. Overall, CAR-T cell responses in the bridging RT and non-bridging RT groups were 80 and 64%, respectively. Lastly, Imber et al. presented their retrospective analysis of 11 patients with DLBCL or transformed follicular lymphoma who received bridging radiation prior to axi-cel (*n* = 6), JCAR017 (*n* = 3), tisa-cel (*n* = 1), or EGFRt/19-28z/4-1BBL CAR (*n* = 1) (28). The most common RT regimen was 20Gy in 5 fractions (*n* = 6). Local control was excellent but most (*n* = 7) had PD out of field prior to CAR infusion. Day 30 ORR was 100%, and of the 5 evaluable patients at day 90, 3 had continued complete metabolic response and 2 had PD (one with relapse in and out of RT treatment field and one primarily out of field). RT did not seem to increase grade 3 or higher toxicities from CAR-T.

### RT Timing

Emerging data has suggested that oncologic therapies can impact the health and function of the autologous T-cells utilized for production of the CAR T-cell construct. T-cell fitness has been shown to be important for CAR T efficacy. CAR T composition and polyfunctionality was associated with both response and increased toxicity with a greater percentage of effector T-cells in responders vs. non-responders (29). Higher proportions of cycling CD4 T-cells and memory CD8 T-cells were associated with superior clinical response (30). Therapies that are likely to cause prolonged cytopenias, particularly in a patient who is older or less fit, could potentially have a greater negative effect on T-cell fitness. For instance, additional cycles of chemotherapy in patients with acute lymphoblastic leukemia, non-Hodgkin lymphoma (NHL), Hodgkin lymphoma, and acute myelogenous leukemia (AML) deplete naïve, effector memory T-cells and reduce T-cell proliferation capability (31). CAR T-cell fitness also varied by the number of prior lines of therapy received in the ZUMA-1 trial. Median CAR area under the curve (AUC) at Day 0–28 was substantially lower in patients who received 5 or more lines of prior therapy (11). Interestingly in an interim analysis of the ZUMA-12 trial that evaluated axi-cel therapy in the frontline setting for patients with high risk LBCL, the median peak CAR T cell levels and the median CAR T cell expansion levels were greater in the ZUMA-12 patient cohort as compared to the ZUMA-1 cohort (32). These observations suggest that exposure to multiple oncologic therapies can adversely impact the function of autologous cells used for CAR T-cell production. Bendamustine in particular may adversely affect T-cell numbers and function (33, 34).

The optimal timing of RT administration for patients that will undergo CAR T-cell therapy is currently unknown, however oncologists should ideally aim to deliver RT after apheresis. Caution should be exercised when RT is administered prior to T-cell collection. Even when limited RT fields that minimize bone marrow exposure are employed, RT has the potential to adversely impact circulating blood cells. Modeling studies have demonstrated that a single 2 Gy fraction of RT administered for a typical glioblastoma plan to the brain would deliver 0.5 Gy to 5% of the circulating blood cells and after 30 fractions, 99% of the circulating blood could receive at least 0.5 Gy (35). Lymphocytes are highly radiosensitive such that these low dose exposures could reduce lymphocyte counts and impair cell collection. Most importantly however, T-cell function could be impacted by even low dose RT, with subsequent effects on the autologous CAR T cell product that may impact treatment efficacy. However, while we generally recommend radiation after apheresis if possible to avoid impacting circulating T cells prior to apheresis, emerging evidence shows that local irradiation is not inherently immunosuppressive and large proportions of intratumoral T cells can survive clinically relevant doses of radiation (36). These tissue-resident memory T cells may be more radioresistant than circulating T cells and can mediate tumor control.

### RT Target and Dose

Overall, while the early data regarding RT bridging therapy is encouraging, the current data available include studies with limited patient numbers. Additional clinical validation and prospective studies are needed, particularly with regard to questions of optimal radiation dose and target. In the MDACC study, there was a trend toward improved PFS among patients treated with “comprehensive” RT that encompassed all known active sites of disease, compared to patients treated with “focal” RT with active lymphoma excluded from the RT field (25). Indeed, in that study, several patients treated with focal RT experienced relapse in sites that were active at the time of axi-cel infusion and not included in the bridging RT field. Comprehensive radiation may be most compelling for patients with disease in a contiguous or limited region(s) that can be safely encompassed within a radiation field without significant normal tissue toxicity (37). While the optimal dose of radiation is under investigation, early evidence indicates that hypofractionated radiation in clinical and pre-clinical settings can avoid lymphopenia and also result in recruitment of dendritic cells, priming of antitumoral CD8 T cells, and relatively low number of infiltrating regulatory T cells and thus may give us an early rationale for considering hypofractionation over conventional fractionation (38–40). To summarize the practical considerations when considering RT bridging, ideally RT should be delivered after apheresis if possible to minimize impact on T cell fitness, more comprehensive RT treatment may be helpful if it can be delivered safely with minimal toxicity, hypofractionated regimens can often be delivered safely and may result in a more favorable immune microenvironment, and minimizing toxicity that may require steroid treatment is advised.

### RT and Tumor Debulking

Decreased tumor burden prior to CAR T-cell infusion is associated with improved efficacy and decreased toxicity in R/R DLBCL (10). The overall response rate to axi-cel in ZUMA-1, as well as durability of response at 1 year, has been directly associated with lower tumor burden (10, 14, 41). Additionally, there was an association between decreased tumor burden and lower rates of treatment-related toxicity (Grade 3 or higher neurologic events and CRS). The relationship between tumor burden and efficacy was also observed in the US Lymphoma CAR T Consortium study of R/R LBCL patients treated with standard-of-care axi-cel, in which high LDH was the most significant predictive variable in multivariate analysis for shorter PFS and OS (26). High tumor burden was also associated with decreased event-free survival among adult ALL patients following CD19 CAR T-cell therapy (42). Finally, among 96 large B-cell lymphoma patients treated at Moffitt Cancer Center with commercially available axi-cel, elevated tumor burden as identified by high metabolic tumor volume (MTV) on PET-CT was associated with significantly shorter PFS and OS (43). These studies support the notion that optimal tumor debulking can improve CAR T outcomes. RT is a useful tool that can facilitate effective tumor debulking, particularly among patients with highly chemorefractory disease, however it is unknown if debulking with RT prior to CART infusion improves outcomes.

## Radiation as Conditioning Therapy

There is early preclinical evidence that low dose radiation induces tumor cell susceptibility to CAR T mediated killing via TRAIL-mediated death (21). Even at ultra-low radiation doses of 1.8 to 2 Gy, ribonucleic acid (RNA) sequencing analysis of radiation-exposed tumors revealed the transcriptional signature of cells highly sensitive to TRAIL-mediated apoptotic death. If tumor cells are sensitized to CAR T-cell mediated killing through enhanced apoptosis, there is a rationale to investigate the potential role of low dose total nodal or total body irradiation or perhaps even targeted radionuclide approaches (44) as part of the CAR T conditioning regimen. This concept has not yet been clinically investigated, however is supported by reports of disease progression following CAR T therapy only in areas that harbored disease before CAR T infusion that were not included in the radiation field (25).

## Radiation Therapy After Car T-Cell Relapse

Radiation is an attractive early salvage option for patients after disease relapse or progression to CAR T-cell therapy, particularly if it potentiates CAR T-cell mediated death. In a case of a patient with R/R multiple myeloma who received steroids and palliative radiation to the spine for cord compression on days 6–20 after B-cell maturation antigen (BCMA) CAR T-cell infusion, there was a peak in T-cell receptor repertoire expansion, as well as interleukin-6 (IL6) and C-reactive protein (CRP), following RT at a time point later than would be expected with CAR T therapy alone (45). The patient had a complete systemic response, and, despite steroids, there was BCMA CAR T-cell persistence, raising the intriguing possibility that RT may influence both the local and distant treatment response. An early retrospective experience of radiation treatment in the salvage setting for non-Hodgkin lymphomas after CAR T-cell therapy shows that this approach may also be effective in aggressive B-cell lymphoma. In a review of 14 patients treated at Memorial Sloan Kettering Cancer Center with salvage radiation post-CAR T progression, Imber et al. reported median OS after RT of 10 months, with 3 patients bridged to allogeneic transplantation and all patients alive without evidence of disease at the time of analysis (46).

Perhaps an even more novel, personalized approach to selecting patients for radiation treatment after CAR T-cell therapy is warranted. A study of early molecular response (EMR) in R/R DLBCL patients treated with axi-cel revealed that patients who achieved an EMR, defined as a >5-fold reduction in measured plasma-derived cell free DNA (cfDNA) as early as day 7 after infusion, had increased durability of response (30). Patients with an EMR had a 75% CR rate at 3 months compared to 0% CR rate at 3 months for those without an EMR. For those patients who fail to achieve an EMR, there may be an opportunity for early radiation treatment in an effort to reduce disease progression in this higher risk patient population. Whether molecular response can be used to help select patients who may benefit from early salvage radiation treatment merits further investigation.

## Conclusions

CAR T-cell therapy has revolutionized the treatment approach to patients with relapsed/refractory hematologic malignancies, however, there remains opportunity for improving outcomes and toxicity. Radiation therapy can play an important role in combined modality treatment for patients undergoing CAR T therapy in various clinical settings. Future research frontiers include investigation of exciting hypotheses including radiation priming of CAR T-cell mediated death, radiation debulking to reduce tumor burden, and selection of patients at high risk of CAR T failure for early radiation salvage.

## Author Contributions

PF and CP conceived and designed the manuscript. All authors contributed to the writing of the manuscript.

## Conflict of Interest

LN: Honorarium: Bayer, Celgene, Genentech, Gilead/Kite, Janssen, Juno, Novartis, TG Therapeutics, and Spectrum Therapeutics; Research: Celgene, Genentech, Janssen, TG Therapeutics, and Karus Therapeutics. SN: Research support from Kite/Gilead, Merck, BMS, Cellectis, Poseida, Karus, Acerta, and Unum Therapeutics. Advisory Board Member/Consultant for Kite/Gilead, Merck, Celgene, Novartis, Unum Therapeutics, Pfizer, Precision Biosciences, Cell Medica, Calibr, Allogene, Incyte, and Legend Biotech. CP: Research support from Merck. The remaining authors declare that the research was conducted in the absence of any commercial or financial relationships that could be construed as a potential conflict of interest.
